# Characterisation of the immune response to type I collagen in scleroderma

**DOI:** 10.1186/ar2025

**Published:** 2006-07-31

**Authors:** Kenneth J Warrington, Usha Nair, Laura D Carbone, Andrew H Kang, Arnold E Postlethwaite

**Affiliations:** 1 Department of Medicine, Division of Connective Tissue Diseases, 956 Court Avenue, Room G326, Memphis, TN 38163; 2 Veterans Affairs Medical Center, Memphis, 1030 Jefferson Avenue, Memphis, TN 38104, USA; 3 Department of Molecular Sciences, University of Tennessee Health Science Center, 956 Court Avenue, Room A318, Memphis, TN 38163 USA

## Abstract

This study was conducted to examine the frequency, phenotype, and functional profile of T lymphocytes that proliferate in response to type I collagen (CI) in patients with scleroderma (SSc). Peripheral blood mononuclear cells (PBMCs) from SSc patients, healthy controls, and rheumatoid arthritis disease controls were labeled with carboxy-fluorescein diacetate, succinimidyl ester (CFSE), cultured with or without antigen (bovine CI) for 14 days, and analysed by flow cytometry. Surface markers of proliferating cells were identified by multi-color flow cytometry. T-cell lines were derived after sorting for proliferating T cells (CFSE^low^). Cytokine expression in CI-responsive T cells was detected by intracellular staining/flow cytometry and by multiplex cytokine bead assay (Bio-Plex). A T-cell proliferative response to CI was detected in 8 of 25 (32%) SSc patients, but was infrequent in healthy or disease controls (3.6%; *p *= 0.009). The proliferating T cells expressed a CD4^+^, activated (CD25^+^), memory (CD45RO^+^) phenotype. Proliferation to CI did not correlate with disease duration or extent of skin involvement. T-cell lines were generated using *in vitro *CI stimulation to study the functional profile of these cells. Following activation of CI-reactive T cells, we detected intracellular interferon (IFN)-γ but not interleukin (IL)-4 by flow cytometry. Supernatants from the T-cell lines generated *in vitro *contained IL-2, IFN-γ, GM-CSF (granulocyte macrophage-colony-stimulating factor), and tumour necrosis factor-α, but little or no IL-4 and IL-10, suggesting that CI-responsive T cells express a predominantly Th1 cytokine pattern. In conclusion, circulating memory CD4 T cells that proliferate to CI are present in a subset of patients with SSc, but are infrequent in healthy or disease controls.

## Introduction

Systemic sclerosis (scleroderma) (SSc) is characterised by immune activation, microvascular dysfunction, and progressive fibrosis. Increased deposition of type I collagen (CI) is evident in the skin and involved internal organs of patients with SSc [1].

Cellular components and soluble mediators of the adaptive immune system play a central role in disease pathogenesis [2]. Activated T cells and levels of soluble interleukin (IL)-2 receptor are increased in the peripheral blood of patients with SSc [3-5]. In the skin, cellular infiltration precedes dermal fibrosis and consists of activated T lymphocytes, plasma cells, and macrophages [6,7]. Helper (CD4) T cells predominate, and the degree of cellular infiltration correlates with both the degree and progression of skin thickening [8]. Memory T cells are also present in the inflammatory infiltrate of affected internal organs, such as the lungs [9].

There is evidence to suggest that the activation of T cells in SSc is antigen-driven [10]. Analysis of the T-cell receptor repertoire in skin biopsies of patients with SSc revealed that T cells have undergone clonal expansion. Indeed, the presence of a dominant T-cell clone in skin biopsies obtained from a patient at different time points and from different skin regions implies that the putative driving antigen is persistently present and widely distributed [11].

Putative antigens in SSc include DNA topoisomerase I, RNA polymerases, and microbial products. CI has also been implicated as an autoantigen in SSc, and several reports suggest that patients with SSc exhibit cellular immunity to CI [12-14]. Peripheral blood mononuclear cells (PBMCs) from the majority of patients produce chemotactic cytokines when cultured with CI [12]. CI-stimulated PBMCs from patients with SSc produce IL-6 [13] and IL-2; the latter is predominantly derived from CD4^+^, but not CD8^+^, T cells [14]. McKown *et al*. reported that PBMCs from most patients with SSc produce IFN-γ, IL-10, or both when cultured with the α chains of CI [15]. Lymphocyte proliferation to CI, measured by tritiated thymidine incorporation, has been reported to occur in a subset (25%) of patients with SSc [12], although this was not confirmed by other investigators [16].

The study of antigen-specific lymphocytes is challenging because these cells are rare in the peripheral blood. Recently, a flow cytometric method that allows the concurrent analysis of the phenotype and proliferative response of antigen-specific T cells was used to study the immune response to a specific antigen [17]. We employed a similar method to demonstrate that, in a subset of patients with SSc but rarely in normal or disease controls, circulating CI-responsive CD4 T cells are present. T cells proliferating in the presence of CI express an activated, memory phenotype and secrete Th1 cytokines.

## Materials and methods

### Research subjects

Patients with a diagnosis of limited or diffuse SSc according to the criteria of the American College of Rheumatology (ACR) (1980) [18] were recruited from the Rheumatology Clinics of the University of Tennessee Health Science Center (Memphis, TN, USA). Patients with SSc-like illness related to environmental, ingested, or injected agents, localised scleroderma, or eosinophilic fasciitis were excluded from the study. Healthy controls were recruited from among staff and allied health workers at the University of Tennessee. Disease controls, all carrying a diagnosis of rheumatoid arthritis according to ACR criteria [19], were recruited from our Rheumatology Clinics. All subjects gave written consent, and the research protocol was approved by the Institutional Review Board.

### Reagents

Bovine CI and β1,2 chain (dimers formed by the component α-1 and α-2 chains) were provided by the Collagen Core facility of the Rheumatic Disease Research Core at the University of Tennessee Health Science Center. Bovine CI was prepared as previously reported [15,20]. The homogeneity of CI was confirmed by sodium dodecyl sulfate-polyacrylamide gel electrophoresis and by cyanogen bromide peptide mapping. Fluorochrome-labeled antibodies specific for human lymphocyte surface markers (CD3, CD4, CD8, CD28, CD45RO, CD25, CD212/IL-12R β2, and CD49a) and human cytokines (IFN-γ and IL-4) were obtained from BD Biosciences (San Jose, CA, USA). Human rIL-2 was obtained from Sigma-Aldrich (St. Louis, MO, USA). Cell culture medium ('complete medium') consisted of RPMI-1640, 2 mM l-Glutamine, 1% non-essential amino acids, 1% sodium pyruvate, 100 U/ml penicillin, 100 μg/ml streptomycin, 25 mM HEPES Buffer, and 55 μM 2-mercaptoethanol (all from Invitrogen, Carlsbad, CA, USA) and 9% fetal calf serum (Sigma-Aldrich).

### PBMC isolation, CFSE labeling, and cell culture

PBMCs were isolated from venous blood by density-gradient centrifugation using Ficoll-Paque (GE Healthcare, Little Chalfont, Buckinghamshire, UK). Cells were immediately labeled with carboxy-fluorescein diacetate, succinimidyl ester (CFSE) (Sigma-Aldrich), using a modification of the method described by Turcanu *et al*. [17]. Briefly, PBMCs were resuspended at 1 × 10^7 ^cells per ml in phosphate-buffered saline (PBS) containing 0.1% bovine serum albumin (BSA) (Sigma-Aldrich). Prior to staining, an aliquot of CFSE (5 mM in dimethyl sulfoxide) was thawed, diluted 1:10 with 1 M HEPES buffer (Invitrogen), and added to the cell suspension (labeling concentration was 5 μM). PBMCs were vortexed and incubated with CFSE for 10 minutes in a 37°C water bath, and the dye excess was washed once with 0.1% BSA/PBS followed by a wash with complete medium. PBMCs were cultured in complete medium in 24-well plates at a concentration of 3 × 10^6^/2 ml medium per well in a 37°C, 5% CO_2 _incubator. CI was added to the cell culture at a final concentration of 10 μg/ml, and β1,2 chain was added in a separate well (final concentration of 2.5 μg/ml). Control wells received only PBS. At day 7, half the medium from all wells was replaced with fresh medium supplemented with IL-2 (20 U/ml). PBMCs were cultured for a total of 12 to 14 days and were then harvested, washed, and analysed by flow cytometry. In the first 13 patients with SSc studied, PBMCs were cultured with CI or PBS control. In the next 12 patients who were enrolled, PBMCs were cultured with CI, purified β1,2 chain, or PBS control. We included β1,2 chain in these subsequent experiments to demonstrate that T-cell proliferation was not due to a collagen contaminant. For all the RA and normal subjects, PBMCs were cultured with CI, purified β1,2 chain, or PBS control. Correlation between the results obtained with CI and β1,2 chain was excellent. (Individual subject PBMCs responded either to both collagen preparations or to neither preparation.)

A positive proliferative response to CI was defined as a greater-than-twofold increase in proliferating T cells (CFSE^low^) in response to antigen.

### Flow cytometry

Surface markers of proliferating cells were identified by multi-color flow cytometry with fluorochrome-labeled antibodies to CD3, CD4, CD8, CD28, CD45RO, CD25, CD212 (IL-12R β2), and CD49a. Briefly, lymphocytes were stained for 30 minutes at 4°C with PerCP- (peridinin-chlorophyll-protein) or PE- (phycoerythrin) conjugated antibodies. Appropriate isotype controls were used to determine background staining. After washing of unbound antibody, samples were analysed on a FACScan flow cytometer (Becton, Dickinson and Company, Franklin Lakes, NJ, USA), and the frequencies of cell subsets were calculated using WinMDI software (Joseph Trotter; The Scripps Research Institute, La Jolla, CA, USA).

### Generation of T-cell lines

CI-responsive T-cell lines were generated from the PBMCs of three patients which demonstrated a positive *in vitro *proliferative response to CI (as defined above). After 14 days of culture with antigen, cells were collected and stained with anti-CD4 PE. CFSE^low^CD4^+ ^cells or CFSE^high^CD4^+ ^cells were then sorted using a FACSCalibur (BD Biosciences) CFSE^low^CD4^+ ^cells were further expanded by two to three rounds (every 10 days) of *in vitro *activation with irradiated (4,000 rad) autologous PBMCs (10^6^/well), antigen (CI or β1,2 chain), and IL-2 (20 U/ml). After the last round of activation, cells were rested for 10 days prior to additional experiments.

### Intracellular cytokine detection by flow cytometry

Resting T-cell lines (10^6^/ml in 48-well plates) were restimulated with PMA (phorbol 12-myristate 13-acetate) (50 ng/ml) and ionomycin (500 ng/ml) in the presence of brefeldin A (10 μg/ml; GolgiPlug; BD Biosciences) for 5 hours. The cells were then harvested, stained with antibodies to surface markers (CD4) for 30 minutes, fixed (20 minutes, 4% paraformaldehyde/PBS), and stored at 4°C overnight. Next, the cells were permeabilised (BD Perm/Wash; BD Biosciences) and then stained with cytokine-specific antibodies at 4°C for 30 minutes. Parallel samples were incubated with appropriate intracellular isotype controls. Samples were run on a flow cytometer, and at least 30,000 events were collected. Data were analysed using the WinMDI 2.8 software (The Scripps Research Institute).

### Cytokine array

In separate experiments, resting CI-specific T-cell lines were activated *in vitro *for 72 hours with plate-bound anti-CD3 (10 μg/ml) and anti-CD28 (1 μg/ml). Cells were incubated at a concentration of 2 × 10^6 ^cells per ml in a 48-well plate, and supernatants were harvested after 48 hours of culture. T-cell line supernatants were then assayed for cytokines using a multiplex cytokine bead array system (cat. no. 171-A11050 Bio-Plex; Bio-Rad, Hercules, CA, USA) according to the manufacturer's instructions. The reaction mixture was read using the Bio-Plex protein array reader, and data were analysed with the Bio-Plex Manager software program in the Rheumatic Disease Research Core Center, Veterans Affairs Medical Center (Memphis, TN, USA).

### Statistical analysis

Descriptive statistics were generated using SigmaStat software (version 2.03; SPSS Inc., Chicago, IL, USA). The proportion of patients and controls demonstrating reactivity to CI was compared using the Fisher exact test (SigmaStat software).

## Results

### Patient demographics

We enrolled 25 patients with SSc for this study. The mean age was 55.4 (± 10.9) years, and 72% of the subjects were female. Most patients had limited cutaneous disease, and eight (32%) had diffuse disease. The average SSc disease duration was 10.29 (± 9.8) years (Table 1).

### CI stimulation results in the expansion of CFSE^low^CD4^+ ^T lymphocytes

**Figure 1:**
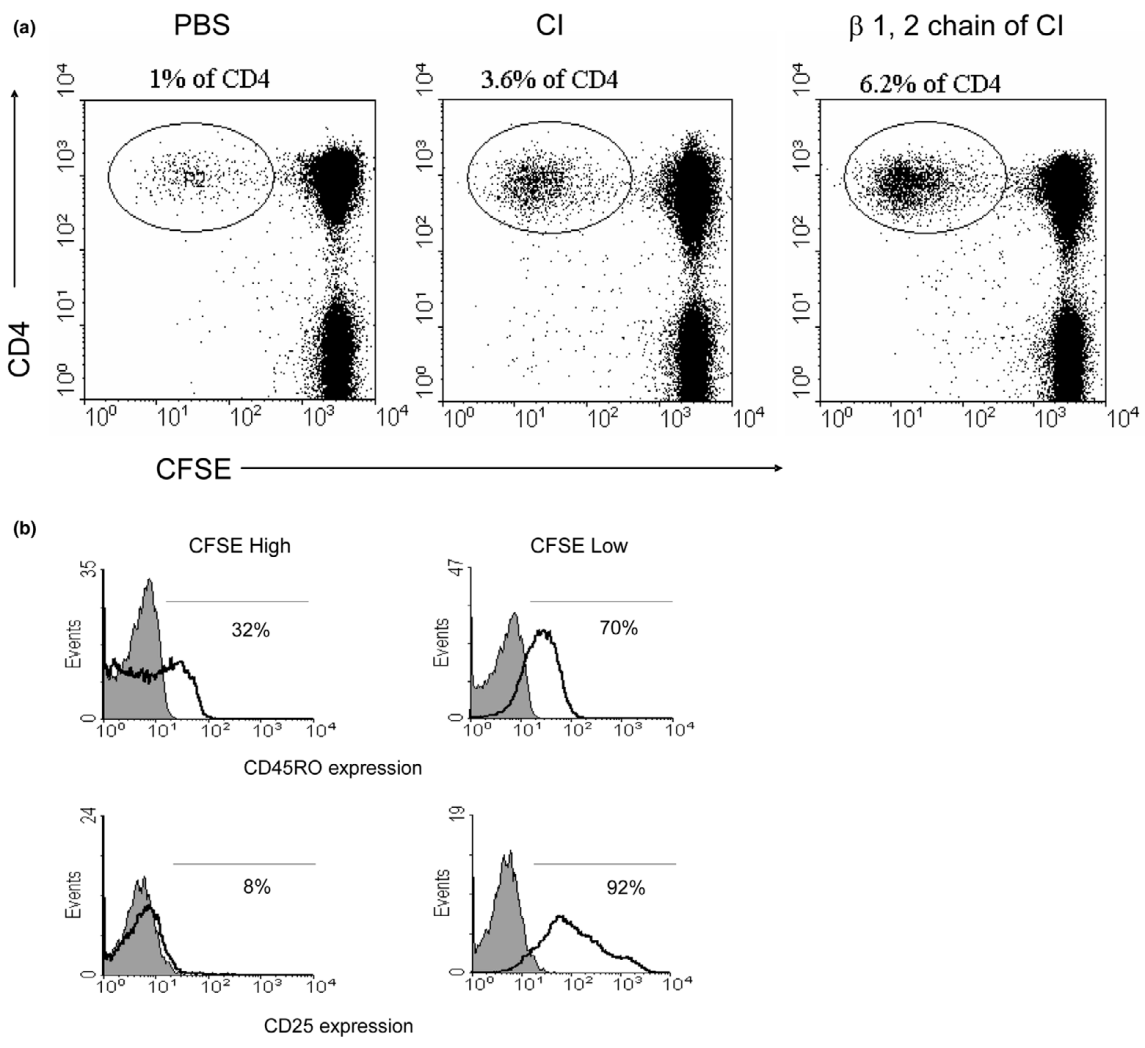
T-Cell Proliferation to C1. (a) Peripheral blood mononuclear cell proliferation monitored using carboxy-fluorescein diacetate, succinimidyl ester (CFSE) labeling. Representative dot plot of CFSE/CD4 double-labeling demonstrates increased T-cell proliferation (CFSE^low^) in response to (*in vitro *culture with) type I collagen (CI) or β1,2 chain. (b) CI-reactive lymphocytes express an activated, memory phenotype. Representative histogram demonstrates that the majority of CFSE^low ^(that is, antigen-specific proliferating T cells) express CD45RO and CD25 (right panels) compared with the CFSE^high ^(non-responsive to antigen) T cells (left panels). Grey shaded areas represent staining with the appropriate isotype control. PBS, phosphate-buffered saline.

PBMCs from SSc donors were labeled with CFSE and then cultured with and without CI. To further expand the CI-specific population, IL-2 was added at day 7 and the cells were harvested at day 14. In the CI-treated cultures, we observed the expansion of a CFSE^low^CD4^+ ^T lymphocyte population. A much smaller population of CFSE^low^CD4^+ ^T lymphocytes was present in the PBMCs cultured without antigen, suggesting that the CFSE^low^population emerges due to antigen-induced proliferation. Other studies have shown that this method correlates very well with cell proliferation measured by [^3^H]-thymidine incorporation [17]. A distinct advantage of this method is that proliferating cells are viable and can be used for additional experiments such as immunophenotyping and functional analyses. To confirm our findings, concurrent experiments were conducted in select patients, using highly purified β1,2 chain (heterodimers of α-1 and α-2 chains) of CI as antigen, and similar results were obtained (Figure 1a). A positive proliferative response to CI was defined as a greater-than-twofold increase in proliferating cells (CFSE^low^) in the presence of CI. Approximately one third of the patients with SSc demonstrated reactivity to CI, whereas CI reactivity was rare in control cohorts (*p *= 0.009). Only one of 19 healthy individuals demonstrated significant T-cell proliferation to CI, and none of the patients with rheumatoid arthritis reacted to CI *in vitro *(Table 2). Among those patients exhibiting a proliferative response to CI, the mean percentage of proliferating CD4 T cells increased from 11% (± 9.97) in control wells to 28.7% (± 19.4) in the CI-stimulated wells (*p *= 0.04).

In the patients with SSc, there was no significant difference in disease duration between the CI responder group (median disease duration 10 years) and the CI non-responders (median disease duration 6 years; *p *= 0.561). There was no correlation between CI responsiveness and pattern of skin involvement (limited versus diffuse; *p *= 0.359). None of the SSc-related disease manifestations was predictive of an immune response to CI *in vitro*, except for a trend toward more lung fibrosis in the CI responders. Of the CI responders, 87.5% had pulmonary involvement, whereas only 47% of the non-responders had this complication (*p *= 0.09). It is possible that in a study with a larger sample size a significant difference would be observed.

Furthermore, we used three-color (CFSE, PE, and PercP) flow cytometry to compare the cell surface characteristics of CI-responsive T cells (CFSE^low^) with the non-proliferating cells (CFSE^high^). In patients who exhibited a significant proliferative response to CI, PBMCs (at day 14 of culture with CI) were stained with fluorochrome-conjugated antibodies to CD3, CD4, CD25, CD45RO, and CD212 (IL-12 receptor β2). Using flow cytometry and by electronically gating on the CFSE^low ^population, we determined that CFSE^low ^cells were CD3^+^CD4^+ ^T cells that preferentially expressed the activation marker CD25 (IL-2 receptor α) and the memory T-cell marker CD45RO. As expected, the CFSE^high^, non-proliferating population included both naïve and memory T cells that were not activated and did not express the IL-2R (Figure 1b). Although CD212 has been implicated as a useful surface marker for Th1-polarised T cells [21], we did not detect expression of this marker on CI-activated, proliferating T cells (data not shown).

### Generation of CI-responsive T-cell lines

**Figure 2:**
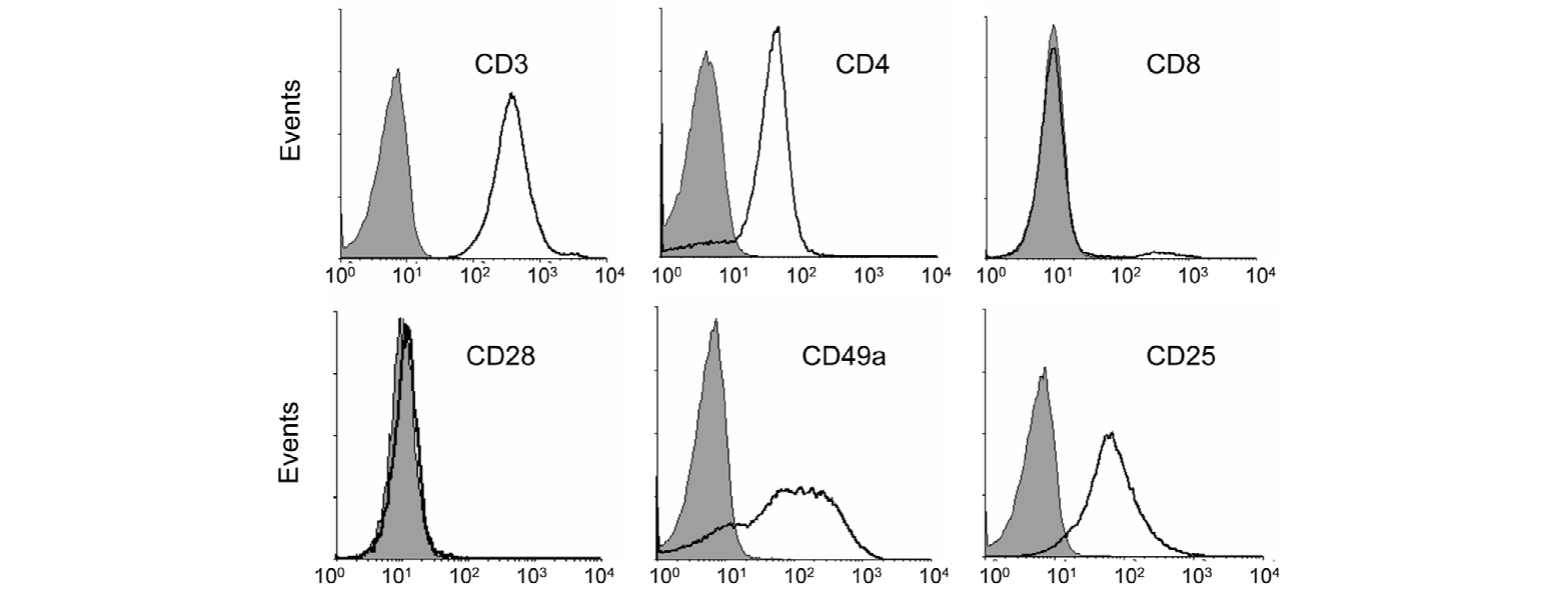
Phenotypic analysis of type I collagen (CI)-responsive T-cell lines. T cells were expanded *in vitro *with autologous peripheral blood mononuclear cells, interleukin-2, and antigen (CI). The resultant T-cell lines that proliferated to CI were CD3^+^CD4^+^CD8^-^CD28^-^CD25^+^CD49a^+^. Grey shaded areas represent staining with the appropriate isotype control.

We generated three CI-activated T-cell lines from three different patients, all exhibiting a T-cell proliferative response to CI (as determined by CFSE staining and flow cytometry). After *in vitro *activation of PBMCs with CI, the CFSE^low ^population (antigen-activated) was sorted by flow cytometry and expanded with autologous, irradiated PBMCs, antigen, and IL-2. All cell lines were CD3^+^CD4^+^CD8^-^CD28^-^CD25^+^CD49a^+^(Figure 2).

### Cell line cytokine profile

**Figure 3:**
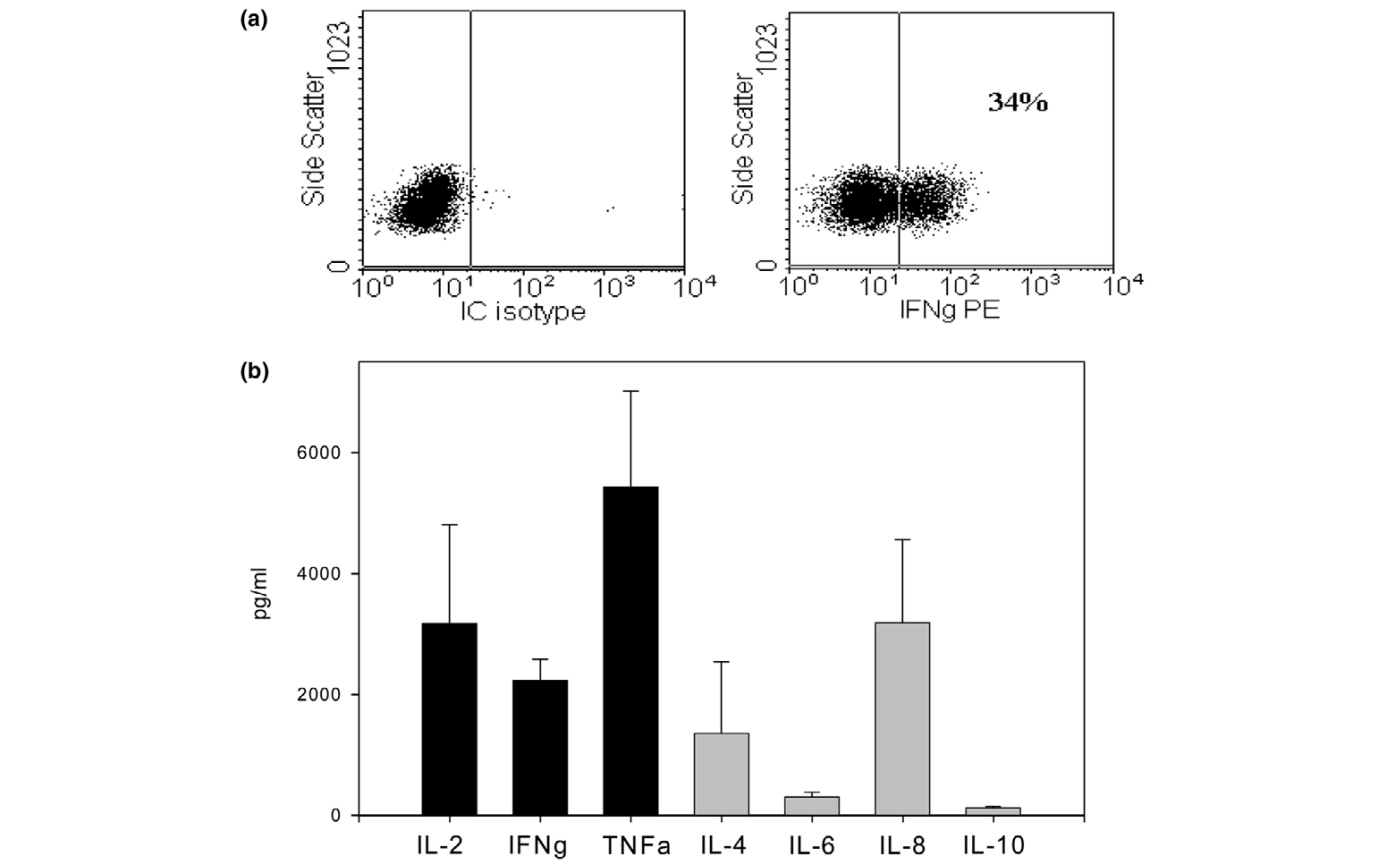
Th1 polarisation of type I collagen (CI)-responsive T-cell lines. Cytokine expression of T-cell lines was determined by intracellular (IC) staining of PMA (phorbol 12-myristate 13-acetate)/ionomycin activated T cells. (a) We detected interferon (IFN)-γ staining but no interleukin (IL)-4 staining. Multiplex cytokine assay was used to analyse the cytokine profile of three T-cell line supernatants. (b) An excess of Th1 over Th2 cytokines was detected. PE, phycoerythrin; TNF, tumour necrosis factor.

We were interested in comparing the Th1 and Th2 cytokine secretion profile of CI-specific cell lines. Cell lines generated from three patients were activated *in vitro *for 72 hours with plate-bound anti-CD3 (10 μg/ml) and anti-CD28 (1 μg/ml). Cell culture supernatants were harvested and assayed for cytokine content using a Bio-Plex assay. We detected abundant Th1 cytokines, including IL-2, IFN-γ, and TNF-α. Th2 cytokines, including IL-4, IL-6, and IL-10, were present in much lower amounts (Figure 3b). The chemotactic cytokine IL-8 was also detected in the supernatant of CI-specific cells and is likely responsible for the chemotactic properties of CI-activated PBMCs in the original publication of Stuart *et al*. [12].

To confirm our findings, we also analysed intracellular cytokine production by flow cytometry, using fluorochrome-conjugated antibodies to IFN-γ and IL-4 as representative cytokines of a Th1 and Th2 profile, respectively. Indeed, intracellular IFN-γ staining, but not IL-4 staining, was detected in our T-cell lines, confirming the Bio-Plex data (Figure 3a).

## Discussion

In this report, we demonstrate that in a subset of patients with SSc, CI may behave as an autoantigen and induce proliferation of a specific CD4 T-cell subset that has a Th1 functional profile.

Our results are consistent with a previous study [12] in which 25% of patients with SSc demonstrated lymphocyte proliferation in response to CI, as measured by [^3^H]-thymidine incorporation. However, previous studies implicating CI as an autoantigen have not examined the phenotype or functional profile of the antigen-specific cells. T cells that recognise a particular antigen are present with very low frequency in the blood, and therefore direct analysis is technically challenging. We used CFSE labeling to allow identification of proliferating, antigen-specific cells after *in vitro *stimulation with antigen [22]. This technique was recently employed to study lymphocyte responses to peanut allergens in children with peanut allergy [17]. The advantage of using CFSE over the traditional [^3^H]-thymidine incorporation method is that antigen-specific cells remain viable, allowing for phenotypic analysis, cell separation, and further expansion. Assessment of lymphocyte proliferation using CFSE/flow cytometry correlates well with proliferation as measured by [^3^H]-thymidine incorporation, and CFSE does not interfere with lymphocyte proliferation or function [17].

Mechanisms resulting in the breakdown of tolerance to CI are as yet unknown. CI is the most abundant of all collagens in humans. It is the most abundant collagen in skin, heart, and intestines, all of which are affected in SSc. CI is a heterotrimer molecule composed of two identical α-1(I) chains and one α-2(I) chain, with each α chain containing 1,014 amino acid residues. In mammals, CI is highly conserved, and there is a 92% homology at the amino acid level between human and bovine CI [23]. It is possible that the immune response to CI plays a role in SSc disease pathogenesis or may be a secondary phenomenon due to epitope spreading later in the disease process. Immunity to CI has been described in other fibrotic conditions, such as idiopathic pulmonary fibrosis [24] and bleomycin-induced pulmonary fibrosis [25]. Cellular immunity to CI may also be representative of a more generalised immune reactivity to extracellular matrix proteins in SSc. Laminin and, less often, type IV collagen can induce proliferation of SSc lymphocytes [16]. Autoantibodies to fibrillin-1, the major component of microfibrils in the extracellular matrix, are present in approximately one third of Caucasian patients with SSc [26]. In addition, both humoral and cellular immunities to elastin have been described in a subset of patients with SSc [27].

The increased T-cell proliferation in response to CI in SSc may be reflective of an altered activation state of PBMCs in this condition. It has been demonstrated that CI can act as a potent co-stimulatory molecule and induces proliferation of *in vitro *activated CD4^+ ^and CD8^+ ^normal human T-cell lines in the absence of antigen-presenting cells (APCs) [28]. Because our experiments did not involve concomitant ligation of the T-cell receptor with anti-CD3 and our experiments were conducted with whole PBMC populations containing appropriate APCs, it is unlikely that our results are due to co-stimulatory properties of CI. However, it is also plausible that animal serum in 'complete medium' may have resulted in non-specific T-cell activation that was augmented in the collagen-stimulated cultures.

The CD4^+ ^phenotype of CI-specific T cells is consistent with murine studies showing that type II collagen-specific T cells in the collagen-induced arthritis mouse model also express a CD4^+ ^phenotype and express high levels of Th1 cytokines [29]. CD28^neg ^expression may reflect autoreactive properties of these cells, as has been demonstrated in other autoimmune diseases [30,31]. However, we cannot exclude the possibility that CD28 loss occurred due to *in vitro *T-cell culture and repeated activation, which is known to result in downregulation of CD28 expression [32]. The expression of CD49a (VLA [very late antigen]-1) on our CI-specific T-cell lines is in agreement with the work of Goldstein *et al*. demonstrating that CD49a^+ ^T cells are a subset of Th1-polarised memory T cells that proliferate in response to recall antigens [33].

The cytokine profile in SSc is skewed to a predominantly Th2 pattern, and this has direct consequences on the progression of fibrosis [34,35]. *In vitro*, IL-4 promotes excess extracellular matrix production [36], and Th2 cytokines *in vivo *are associated with increased lung fibrosis in SSc [37]. IL-10 also correlates with increased disease severity, increased skin thickness, and the presence of pulmonary fibrosis [38]. Interestingly, CI-responsive T cells derived from patients with SSc exhibited a Th1 profile, with a predominance of IL-2, IFN-γ, and TNF-α. When human T cells are repeatedly stimulated *in vitro*, they will often develop a Th2 phenotype [39], and therefore it is unlikely that our results are due to the *in vitro *T-cell culture. However, we cannot exclude the possibility that the IL-2 present in our cultures may have skewed T cells to a Th1 phenotype [40].

Expansion of Th1 T cells *in vivo *may be beneficial to limiting disease progression. Lower serum IFN-γ levels have been associated with active SSc [41]. Also, increased production of IFN-γ by CI-activated PBMCs correlated with higher forced vital capacity (Postlethwaite AE, personal communication), consistent with the known anti-fibrotic properties of IFN-γ [42]. Patients whose bronchoalveolar lavage cells made IFN-γ mRNA but not type 2 cytokines were noted to have preserved forced vital capacity over time [37]. The production of TNF-α by CI-specific T cells may also play a role in modulating the homeostasis of extracellular matrix in SSc. Indeed, TNF-α prevents the transforming growth factor-β-induced upregulation of α-2(I) collagen and tissue inhibitor of metalloproteinases 1 in dermal fibroblasts [43] and directly stimulates the production of matrix metalloproteinase-1 [44]. Our study was limited to a fairly small sample size, making it difficult to perform detailed correlations among disease phenotype, disease progression, and immune responses to CI.

However, the importance of immunity to CI is emphasised by the finding that oral administration of CI to patients with SSc modulates T-cell responses and may ameliorate the disease [15]. Similar findings have been confirmed in a large multi-centre, placebo-controlled, randomised clinical trial that was recently completed [45].

## Conclusion

Circulating, memory CD4 T cells that proliferate in response to CI are present in a subset of patients with SSc but are infrequent in healthy or disease controls. Determining T-cell responses to CI in subsets of patients with SSc over time and using methods employed in our study may help to identify those who are most likely to benefit from CI-based immunotherapy.

## Abbreviations

ACR = American College of Rheumatology; APC = antigen-presenting cell; BSA = bovine serum albumin; CFSE = carboxy-fluorescein diacetate, succinimidyl ester; CI = type I collagen; IFN-γ = interferon-γ ; IL = interleukin; PBMC = peripheral blood mononuclear cell; PBS = phosphate-buffered saline; PE = phycoerythrin; SSc = scleroderma; TNF-α = tumour necrosis factor-α.

## Competing interests

The authors declare that they have no competing interests.

## Authors' contributions

KJW was responsible for the study design, data interpretation, and drafting of the manuscript. UN conducted the flow cytometry studies and analyses. LDC recruited study subjects and assisted with data interpretation and drafting of the manuscript. AHK participated in study design and data interpretation. AEP participated in study design, data interpretation, and manuscript preparation. All authors read and approved the final version.

## Figures and Tables

**Table 1:** Patient demographics

Characteristic	*n *(%)
Mean age (years)	55.4 ± 10.9
Gender	
Male	7 (28%)
Female	18 (72%)
Race	
Caucasian	19 (76%)
African American	6 (24%)
Disease duration (years)	10.29 ± 9.8
Skin involvement	
Diffuse disease	8 (32%)
Mean skin score (MRSS)	24.5 ± 8.2
Limited disease	17 (68%)
Mean skin score (MRSS)	13 ± 5.9
Raynaud's	24 (96%)
Arthralgias	23 (88%)
Internal organ involvement	
Gastrointestinal	24 (96%)
Pulmonary	15 (60%)
Renal	2 (8%)
Cardiac	2 (8%)

MRSS, modified Rodnan skin score.

**Table 2:** Collagen-specific T-cell response

	CI response
Controls (*n*)	1/28 (3.6%)
Healthy (19)	
Rheumatoid arthritis (9)	

SSc	8/25 (32%)
Limited	5/17 (29.4%)
Diffuse	3/8 (37.5%)

Positive proliferative response to type I collagen (CI) was defined as a greater-than-twofold increase in proliferating cells (CFSE^low^) in the presence of CI. Approximately one third of patients with scleroderma (SSc) demonstrate reactivity to CI, which was significantly higher than the proportion of controls (healthy and rheumatoid arthritis combined) responsive to CI (*p *= 0.009).

## References

[B1] Seyer JM, Kang AH, Rodnan G (1981). Investigation of type I and type III collagens of the lung in progressive systemic sclerosis. Arthritis Rheum.

[B2] White B (1996). Immunopathogenesis of systemic sclerosis. Rheum Dis Clin North Am.

[B3] Fiocco U, Rosada M, Cozzi L, Ortolani C, De Silvestro G, Ruffatti A, Cozzi E, Gallo C, Todesco S (1993). Early phenotypic activation of circulating helper memory T cells in scleroderma: correlation with disease activity. Ann Rheum Dis.

[B4] Freundlich B, Jimenez SA (1987). Phenotype of peripheral blood lymphocytes in patients with progressive systemic sclerosis: activated T lymphocytes and the effect of D-penicillamine therapy. Clin Exp Immunol.

[B5] Steen VD, Engel EE, Charley MR, Medsger TA (1996). Soluble serum interleukin 2 receptors in patients with systemic sclerosis. J Rheumatol.

[B6] Prescott RJ, Freemont AJ, Jones CJ, Hoyland J, Fielding P (1992). Sequential dermal microvascular and perivascular changes in the development of scleroderma. J Pathol.

[B7] Fleischmajer R, Perlish JS, Reeves JR (1977). Cellular infiltrates in scleroderma skin. Arthritis Rheum.

[B8] Roumm AD, Whiteside TL, Medsger TA, Rodnan GP (1984). Lymphocytes in the skin of patients with progressive systemic sclerosis. Quantification, subtyping, and clinical correlations. Arthritis Rheum.

[B9] Wells AU, Lorimer S, Majumdar S, Harrison NK, Corrin B, Black CM, Jeffery PK, du Bois RM (1995). Fibrosing alveolitis in systemic sclerosis: increase in memory T-cells in lung interstitium. Eur Respir J.

[B10] Sakkas LI, Platsoucas CD (2004). Is systemic sclerosis an antigen-driven T cell disease?. Arthritis Rheum.

[B11] Sakkas LI, Xu B, Artlett CM, Lu S, Jimenez SA, Platsoucas CD (2002). Oligoclonal T cell expansion in the skin of patients with systemic sclerosis. J Immunol.

[B12] Stuart JM, Postlethwaite AE, Kang AH (1976). Evidence for cell-mediated immunity to collagen in progressive systemic sclerosis. J Lab Clin Med.

[B13] Gurram M, Pahwa S, Frieri M (1994). Augmented interleukin-6 secretion in collagen-stimulated peripheral blood mononuclear cells from patients with systemic sclerosis. Ann Allergy.

[B14] Hawrylko E, Spertus A, Mele CA, Oster N, Frieri M (1991). Increased interleukin-2 production in response to human type I collagen stimulation in patients with systemic sclerosis. Arthritis Rheum.

[B15] McKown KM, Carbone LD, Bustillo J, Seyer JM, Kang AH, Postlethwaite AE (2000). Induction of immune tolerance to human type I collagen in patients with systemic sclerosis by oral administration of bovine type I collagen. Arthritis Rheum.

[B16] Huffstutter JE, DeLustro FA, LeRoy EC (1985). Cellular immunity to collagen and laminin in scleroderma. Arthritis Rheum.

[B17] Turcanu V, Maleki SJ, Lack G (2003). Characterization of lymphocyte responses to peanuts in normal children, peanut-allergic children, and allergic children who acquired tolerance to peanuts. J Clin Invest.

[B18] (1980). Preliminary criteria for the classification of systemic sclerosis (scleroderma). Subcommittee for scleroderma criteria of the American Rheumatism Association Diagnostic and Therapeutic Criteria Committee. Arthritis Rheum.

[B19] Arnett FC, Edworthy SM, Bloch DA, McShane DJ, Fries JF, Cooper NS, Healey LA, Kaplan SR, Liang MH, Luthra HS (1988). The American Rheumatism Association 1987 revised criteria for the classification of rheumatoid arthritis. Arthritis Rheum.

[B20] Seyer JM, Hutcheson ET, Kang AH (1977). Collagen polymorphism in normal and cirrhotic human liver. J Clin Invest.

[B21] Rogge L, Papi A, Presky DH, Biffi M, Minetti LJ, Miotto D, Agostini C, Semenzato G, Fabbri LM, Sinigaglia F (1999). Antibodies to the IL-12 receptor beta 2 chain mark human Th1 but not Th2 cells *in vitro* and *in vivo*. J Immunol.

[B22] Lyons AB, Parish CR (1994). Determination of lymphocyte division by flow cytometry. J Immunol Methods.

[B23] Miller EJ, Piez KA, Reddi AH (1984). Chemistry of the collagens and their distribution. Extracellular Matrix Biochemistry.

[B24] Kravis TC, Ahmed A, Brown TE, Fulmer JD, Crystal RG (1976). Pathogenic mechanisms in pulmonary fibrosis: collagen-induced migration inhibition factor production and cytotoxicity mediated by lymphocytes. J Clin Invest.

[B25] Schrier DJ, Phan SH, Ward PA (1982). Cellular sensitivity to collagen in bleomycin-treated rats. J Immunol.

[B26] Tan FK, Arnett FC, Antohi S, Saito S, Mirarchi A, Spiera H, Sasaki T, Shoichi O, Takeuchi K, Pandey JP (1999). Autoantibodies to the extracellular matrix microfibrillar protein, fibrillin-1, in patients with scleroderma and other connective tissue diseases. J Immunol.

[B27] Daskalova M, Taskov H, Dimitrova E, Baydanoff S (1997). Humoral and cellular immune response to elastin in patients with systemic sclerosis. Autoimmunity.

[B28] Rao WH, Hales JM, Camp RD (2000). Potent costimulation of effector T lymphocytes by human collagen type I. J Immunol.

[B29] Latham KA, Whittington KB, Zhou R, Qian Z, Rosloniec EF (2005). Ex vivo characterization of the autoimmune T cell response in the HLA-DR1 mouse model of collagen-induced arthritis reveals long-term activation of type II collagen-specific cells and their presence in arthritic joints. J Immunol.

[B30] Markovic-Plese S, Cortese I, Wandinger KP, McFarland HF, Martin R (2001). CD4^+ ^CD28^- ^costimulation-independent T cells in multiple sclerosis. J Clin Invest.

[B31] Schmidt D, Goronzy JJ, Weyand CM (1996). CD4^+ ^CD7^- ^CD28^- ^T cells are expanded in rheumatoid arthritis and are characterized by autoreactivity. J Clin Invest.

[B32] Vallejo AN, Brandes JC, Weyand CM, Goronzy JJ (1999). Modulation of CD28 expression: distinct regulatory pathways during activation and replicative senescence. J Immunol.

[B33] Goldstein I, Ben-Horin S, Li J, Bank I, Jiang H, Chess L (2003). Expression of the α1β1 integrin, VLA-1, marks a distinct subset of human CD4^+ ^memory T cells. J Clin Invest.

[B34] Atamas SP, White B (1999). Interleukin 4 in systemic sclerosis: not just an increase. Clin Diagn Lab Immunol.

[B35] Hasegawa M, Fujimoto M, Kikuchi K, Takehara K (1997). Elevated serum levels of interleukin 4 (IL-4), IL-10, and IL-13 in patients with systemic sclerosis. J Rheumatol.

[B36] Postlethwaite AE, Holness MA, Katai H, Raghow R (1992). Human fibroblasts synthesize elevated levels of extracellular matrix proteins in response to interleukin 4. J Clin Invest.

[B37] Atamas SP, Yurovsky VV, Wise R, Wigley FM, Goter Robinson CJ, Henry P, Alms WJ, White B (1999). Production of type 2 cytokines by CD8^+ ^lung cells is associated with greater decline in pulmonary function in patients with systemic sclerosis. Arthritis Rheum.

[B38] Sato S, Hasegawa M, Takehara K (2001). Serum levels of interleukin-6 and interleukin-10 correlate with total skin thickness score in patients with systemic sclerosis. J Dermatol Sci.

[B39] Demeure CE, Yang LP, Byun DG, Ishihara H, Vezzio N, Delespesse G (1995). Human naive CD4 T cells produce interleukin-4 at priming and acquire a Th2 phenotype upon repetitive stimulations in neutral conditions. Eur J Immunol.

[B40] Cohen SB, Webb LM, Feldmann M (1996). The method of deriving human T-cell clones alters the proportion of IL-10-producing cells. Immunology.

[B41] Molteni M, Della Bella S, Mascagni B, Bazzi S, Zulian C, Compasso S, Lessi M, Scorza R (1999). Increased interferon-gamma (IFN-γ) levels produced *in vitro* by alloactivated T lymphocytes in systemic sclerosis and Raynaud's phenomenon. Clin Exp Immunol.

[B42] Duncan MR, Berman B (1985). Gamma interferon is the lymphokine and beta interferon the monokine responsible for inhibition of fibroblast collagen production and late but not early fibroblast proliferation. J Exp Med.

[B43] Yamane K, Ihn H, Asano Y, Jinnin M, Tamaki K (2003). Antagonistic effects of TNF-α on TGF-β signaling through down-regulation of TGF-β receptor type II in human dermal fibroblasts. J Immunol.

[B44] Rekdal O, Osterud B, Svendsen JS, Winberg JO (1996). Evidence for exclusive role of the p55 tumor necrosis factor (TNF) receptor in mediating the TNF-induced collagenase expression by human dermal fibroblasts. J Invest Dermatol.

[B45] Postlethwaite AE, Furst DE, Wong WK, Clements P (2005). Oral tolerance (OT) induction to type I collagen (CI) significantly reduces the skin score in patients with diffuse systemic sclerosis (SSc) with late-phase disease. Results of a NIAMS/NIAID multicenter phase II placebo-controlled double blind clinical trial. Arthritis Rheum.

